# Nutritional risk and a high NRS2002 score are closely related to disease progression and poor prognosis in patients with COVID-19

**DOI:** 10.3389/fnut.2023.1089972

**Published:** 2023-04-12

**Authors:** Yuanyuan Zhou, Yi Chen, Xinyi Zhang, Bennan Zhao, Fengjiao Gao, Xiaoyan Yuan, Yanfeng Zhu, Dafeng Liu

**Affiliations:** ^1^Department of Internal Medicine, Public Health and Clinical Center of Chengdu, Chengdu, China; ^2^School of Public Health, Chengdu Medical College, Chengdu, China; ^3^Department of Endocrinology and Metabolism, West China Hospital, Sichuan University, Chengdu, China

**Keywords:** coronavirus disease 2019 (COVID-19), nutritional risk, NRS2002 score, disease progression, prognosis

## Abstract

**Background:**

Organism can lead to excessive nutrient consumption in the infected state and increase nutritional risk, which is detrimental to the control of the infection and can further aggravate the disease.

**Objectives:**

To investigate the impact of nutritional risk and the NRS2002 score on disease progression and prognosis in patients with COVID-19.

**Methods:**

This was a retrospective cohort study including 1,228 COVID-19 patients, who were divided into a with-nutritional risk group (patients with NRS2002 score ≥ 3) and a without-nutritional risk group (patients with NRS2002 score < 3) according to the NRS2002 score at admission. The differences in clinical and outcome data between the two groups were compared, and the relationship between the NRS2002 score and the disease progression and prognosis of COVID-19 patients was assessed.

**Results:**

Of 1,228 COVID-19 patients, including 44 critical illness patients and 1,184 non-critical illness patients, the rate of harboring nutritional risk was 7.90%. Compared with those in the without-nutritional risk group, patients in the with-nutritional risk group had a significantly longer coronavirus negative conversion time, significantly lower serum albumin (ALB), total serum protein (TP) and hemoglobin (HGB) at admission, discharge or 2 weeks, a significantly greater proportion with 3 or more comorbidities, and a significantly higher rate of critical illness and mortality (all *p* < 0.001). Multiple regression analysis showed that nutritional risk, NRS2002 score and ALB at admission were risk factors for disease severity. In addition, nutritional risk, NRS2002 score and TP at admission were risk factors for prognosis. The NRS2002 score showed the best utility for predicting critical illness and death in COVID-19 patients.

**Conclusion:**

Nutritional risk and a high NRS2002 score are closely related to disease progression and poor prognosis in COVID-19 patients. For patients with NRS2002 score > 0.5, early intervention of malnutrition is needed to reduce the occurrence of critical disease. Additionally, for patients with NRS2002 score > 5.5, continuous nutritional support therapy is needs to reduce mortality and improve prognosis.

**Clinical Trial registration**: [https://www.chictr.org.cn/historyversionpub.aspx?regno=ChiCTR2000034563], identifier [Chinese Clinical Trial Register ChiCTR2000034563].

## 1. Introduction

A global pandemic of coronavirus disease 2019 (COVID-19), caused by severe acute respiratory syndrome coronavirus 2 (SARS-CoV-2), poses a serious threat to global health (1). Since the World Health Organization declared COVID-19 an epidemic on March 11, 2020 (2), the number of confirmed cases has increased daily, as many as 676,363,591 confirmed cases and 6,879,900 deaths as of March 8, 2023 (3).

Patients infected with COVID-19 are in a state of catabolic stress and may exhibit varying degrees of systemic inflammation, possibly even a “cytokine storm,” resulting in severe complications such as acute respiratory distress syndrome, metabolic acidosis, septic shock, and multi-organ failure (4, 5). Previous studies have indicated that patients have a higher nutritional risk in stressful conditions such as infection, and the higher the nutritional risk is, the greater the inflammatory response (6, 7). The likely mechanism is that higher nutritional risk may cause an imbalance between energy intake and expenditure of the organism, increasing susceptibility to infection and the ability of viruses to infect T cells, leading to T cell activation and differentiation, production of cytokines from various T cell subsets and subsequent massive release of cytokines involved in the amplification of the inflammatory response, creating a cytokine storm that lead to high-grade inflammation (8, 9). Since the 1918 influenza pandemic and in other viral pneumonias, including the H1N1 virus, disease severity and mortality have been correlated with nutritional status (10, 11). COVID-19 patients, especially those in critical condition, are at risk for malnutrition (12, 13). There are many causes of malnutrition in COVID-19. Fever is the most common symptom in patients with COVID-19, which increases energy expenditure (14). Decreased appetite in COVID-19 patients can affect nutrition intake. In addition, diarrhea is another common symptom of COVID-19 that can lead to malabsorption of nutrients (15). Furthermore, the psychological stress of illness increases the risk of anxiety and depression, further affecting food intake (16). Thus, reasonable tools need to be used in COVID-19 patients for nutritional risk as soon as possible and to provide reasonable nutritional support in patients with high nutritional risk to effectively improve the nutritional status and clinical outcomes. The European Society for Clinical Nutrition and Metabolism (ESPEN) recommends Nutrition Risk Screening 2002 (NRS2002) as a nutritional screening tool for general inpatients (17). For hospitalized COVID-19 patients, previous literature has validated the feasibility of NRS2002 for nutritional risk screening (18, 19). Therefore, this study aims to assess the association of nutritional risk and the NRS2002 score with the disease progression and prognosis of COVID-19 patients.

## 2. Methods

### 2.1. Subjects

This was a retrospective cohort study. All 1,228 patients with COVID-19 from the hospital isolation ward who presented to the Public Health Clinical Centre of Chengdu from January 16, 2020, to January 30, 2022, were retrospectively recruited. The study was approved by the Public and Health Clinical Centre of Chengdu Ethics Committee (ethics approval number: PJ-K2020-26-01). For emerging infectious diseases, the Ethics Commission of the designated hospital waived written informed consent. According to the current national epidemic prevention policy, all COVID-19 patients (including asymptomatic infection, light) are required to be isolated and observed in hospitals.

### 2.2. Inclusion and exclusion criteria

The inclusion criteria were as follows: no sex limit; age ≥18 years; COVID-19; inpatient isolation and treatment time >1 day.

The exclusion criteria were as follows: age <18 years, inpatient isolation and treatment time ≤1 day.

### 2.3. Disease diagnosis, clinical typing and cure criteria

The criteria of disease diagnosis, clinical typing and cure in COVID-19 patients were based on the seventh Trial Version of the Novel Coronavirus Pneumonia Diagnosis and Treatment Guidance (5).

The diagnosis criteria were cases with one of the following etiological pieces of evidence: real-time fluorescence reverse transcription-polymerase chain reaction (RT-PCR) detected the positive nucleic acid of the new coronavirus; sequencing of viral genes.

Clinical types were divided into five types, including asymptomatic infection, light, common, severe and critical illness five types. The typing criteria were as follows: (1) asymptomatic infection indicated that there were no clinical symptoms and no pneumonia manifestations on imaging; (2) the light type indicated that the clinical symptoms were mild, and there were no pneumonia manifestations on imaging; (3) the common type indicated that the clinical symptoms included fever and respiratory tract, and pneumonia could be seen on imaging; (4) the severe type indicated that the patients had any of the following criteria: respiratory distress, RR ≥ 30 times/min; in the resting state, oxygen saturation ≤ 93%; arterial blood oxygen partial pressure (PaO2)/oxygen concentration (FiO2) ≤ 300 mmHg (1 mmHg = 0.133 kPa), living in areas with high altitude (over 1000 meters above sea level), and PaO2/FiO2 should be corrected according to the following formula: PaO2/FiO2*[atmospheric pressure(mmHg)/760]; pulmonary imaging showed that lesions with significant progress over 50% within 24–48 h were managed as heavy; (5) the critical illness type criteria included one of the following conditions: respiratory failure occurs and mechanical ventilation is needed; Shock occurs; and combining other organ failure requires intensive care units (ICU) monitoring.

The cured discharge standard was as follows: the body temperature returned to normal for more than 3 days; respiratory symptoms improved significantly; lung imaging showed a significant improvement in acute exudative lesions; and two consecutive sputum, nasopharyngeal swabs and other respiratory specimens tested negative for nucleic acid (sampling time at least 24 h apart).

### 2.4. Grouping standards

Among the 1,228 COVID-19 cases, 1131 and 97 cases were divided into the without-nutritional risk group (patients with NRS2002 score < 3) and the with-nutritional risk group (patients with NRS2002 score ≥ 3) according to the NRS2002 score at admission.

Among the 1,228 COVID-19 cases, 1,184 non-critical patients (patients with asymptomatic infection, with light and with common clinical type) were assigned to the non-critical group, and 44 critical patients (patients with severe and with critical illness clinical type) were assigned to the critical group.

Among the 1,228 COVID-19 cases, 1223 surviving patients were assigned to the survive group, and 5 dead patients were assigned to the death group.

### 2.5. Nutritional risk screening tool

Nutritional risk was assessed within 48 h of admission by using NRS2002, which consists of three parts, including nutritional status assessment (based on weight loss, body mass index (BMI) and food intake), disease severity (stress metabolism due to the degree of disease) and age (whether greater than or equal to 70 years old). The score ranges from 0 to 7 points. Patients were reclassified as “with nutritional risk” with a score of ≥3, whereas a score <3 indicated “without nutritional risk.” Body weight and height was measured by nurses, food intake and weight loss were recorded by residents, and nutritional screening was first performed by a trained nurse and then reviewed by a clinical nutritionist.

### 2.6. Definition of the viral negative conversion time, disease severity and prognosis

The disease severity included critical illness (COVID-19 patients with severe or critical illness clinic type) and non-critical illness (COVID-19 patients with asymptomatic infection, light or common clinic type).

The prognosis included death and survive within 4 weeks after admission. The coronavirus negative conversion time was the time from onset to the first negative nucleic acid test meeting the discharge criteria.

### 2.7. Data collection

All data of 1,228 cases, including clinical data, laboratory data, demographic data, and NRS2002 score, were collected to establish databases. Researchers strictly controlled the accuracy, completeness and authenticity of all data.

### 2.8. Statistical analyses

SPSS 26.0 (SPSS, Chicago, IL, USA) and GraphPad Prism 8 (GraphPad, CA, USA) were used for statistical analyses. Continuous data with a normal distribution were presented as the mean ± standard deviation, and continuous data with a non-normal distribution were presented as the median and interquartile range (IQR). Enumeration data were expressed in terms of percentage or proportion. Data with a normal distribution and homogeneity of variance between multiple groups were compared using one-way or two-way ANOVA, and further comparison between two groups was performed by using the least significant difference (LSD) *t* test. Data with a normal distribution and homogeneity of variance between two groups were compared by using the independent samples *t* test. Data without a normal distribution and homogeneity of variance between groups were analyzed by using the Wilcoxon rank-sum test. Chi-square tests were used in the enumeration data analysis. Two-factor correlation analysis was performed by using Spearman correlation analysis. The multiple stepwise regression method was used for multi-factor correlation analysis. Receiver operating characteristic (ROC) analysis was used to assess the capacity of the NRS2002 score to distinguish non-critical from critical and surviving COVID-19 patients. A *p* < 0.05 was considered statistically significant.

### 2.9. Patient and public Involvement

Patients and the public were not involved in the development of the research questions or in the design of the study. Patients received verbal and written information about the study. Additionally, the burden of the intervention was assessed by the investigators. The participants were assessed for eligibility, and data collection was performed. Dissemination of the general results (without personally identifying data) will occur on demand.

## 3. Results

### 3.1. Characteristics of the study population

A total of 1,228 patients with COVID-19 were included in this study. Their demographic and clinical characteristics are listed in Table 1. The median age of the enrolled patients was 37 years, and the majority of patients were male (78.83%). The median coronavirus negative conversion time was 11.5 days, and the duration of hospitalization was 15.0 days.

**Table 1:** Baseline characteristics of COVID-19 patients.

Variables	Total (*n* = 1,228)	Range
Male, *n* (%)	968 (78.83)	
Age(year), [*M* (IQR)]	37.0 (30.0–38.0)	18 ~ 87
Disease severity		
Non-critical illness, *n* (%)	1,184 (96.42)	
Critical illness, *n* (%)	44 (3.58)	
Number of comorbidities		
0, *n* (%)	340 (27.69)	
1, *n* (%)	342 (27.85)	
2, *n* (%)	228 (18.56)	
3 or more, *n* (%)	318 (25.90)	
Clinical type of COVID-19		
Asymptomatic, *n* (%)	392 (31.92)	
Light, *n* (%)	137 (11.16)	
Common, *n* (%)	655 (53.34)	
Severe, *n* (%)	25 (2.03)	
Critical, *n* (%)	19 (1.55)	
Source of cases		
Domestically transmitted cases, *n* (%)	157 (12.79)	
Imported cases, *n* (%)	1071 (87.21)	
Prognosis		
Survive, *n* (%)	1223 (99.59)	
Death, *n* (%)	5 (0.41)	
Nutritional risk		
Without (NRS2002 < 3), *n* (%)	1131 (92.10)	
With (NRS2002 ≥ 3), *n* (%)	97 (7.90)	
The coronavirus negative conversion time (day), [*M* (IQR)]	11.5 (8.0–20.0)	2 ~ 89
Duration of hospitalization (day), [*M* (IQR)]	15.0 (12.0–20.0)	3 ~ 91
ALB at admission (g/L), [*M* (IQR)]	43.9 (41.5–46.0)	27.3 ~ 55.3
TP at admission (g/L), [*M* (IQR)]	71.7 (67.9–75.6)	38.2 ~ 92.2
HGB at admission (g/L), [*M* (IQR)]	151.0 (138.0–159.0)	54 ~ 191

NRS2002, nutritional risk screening 2002; ALB, albumin; TP, total protein; HGB, hemoglobin. Continuous variables were presented as median (IQR), categorical variables were showed as *n* (%).

In addition, most of patients had comorbidities, 342 (27.85%) patients had one comorbidity, 228 (18.56%) patients had two comorbidities, 318 (25.90%) patients had three or more comorbidities, and 340 (27.69%) patients had no comorbidities. Among them, 44 (3.58%) patients had critical illness, 1,184 (96.42%) patients had non-critical illness, and 5 (0.41%) patients died.

For the clinical type of COVID-19, there were 31.92% asymptomatic infection, 11.16% light, 53.34% common, 2.03% severe, and 1.55% critical illness. Of them, 12.79% of patients were domestically transmitted cases, and 87.21% were imported cases. According to the NRS2002 assessment, 7.90% of patients had nutritional risk，and 92.10% of patients had no nutritional risk. Furthermore, at admission the median ALB level was 43.9 g/L, the TP level was 71.7 g/L, and the HGB level was 151.0 g/L.

### 3.2. Comparisons between the with nutritional risk group and the without nutritional risk group

Compared with the without-nutritional risk group, the proportion of three or more comorbidities, domestically transmitted cases rate, critical illness rate, and mortality rate were significantly higher in the with-nutritional risk group (Table 2) (all *p* < 0.05).

**Table 2:** Comparison of baseline conditions between two groups (*n* = 1,228).

Variables	NRS group (*n* = 1,228)		*χ^2^*	*P*
	NRS2002 ≥ 3	NRS2002 < 3		
	(*n* = 97)	(*n* = 1131)		
Male, *n* (%)	47 (48.45)	921(81.43)	58.22	<0.001
Number of comorbidities			19.19	<0.001
0, *n* (%)	31 (31.96)	309 (27.32)		
1, *n* (%)	17 (17.52)	325 (28.74)		
2, *n* (%)	9 (9.28)	219 (19.36)		
3 or more, *n* (%)	40 (41.24)	278 (24.58)		
Prognosis			58.54	<0.001
Survive, *n* (%)	92 (94.85)	1131 (100)		
Death, *n* (%)	5 (5.15)	0 (0)		
Disease severity			301.89	<0.001
Critical illness, *n* (%)	34 (35.05)	10 (0.88)		
Non-critical illness, *n* (%)	63 (64.95)	1121 (99.12)		
Source of cases			82.1	<0.001
Domestically transmitted cases, *n* (%)	41 (42.27)	116 (10.26)		
Imported cases, *n* (%)	56 (57.73)	1015 (89.74)		
Clinical type of COVID-19			327.84	<0.001
Asymptomatic, *n* (%)	26 (26.80)	366(32.36)		
Light, *n* (%)	9 (9.38)	128(11.32)		
Common, *n* (%)	28 (28.87)	627(55.44)		
Severe, *n* (%)	15 (15.46)	10 (0.88)		
Critical, *n* (%)	19 (19.59)	0 (0)		

NRS2002, nutritional risk screening 2002.

**Figure 1:**
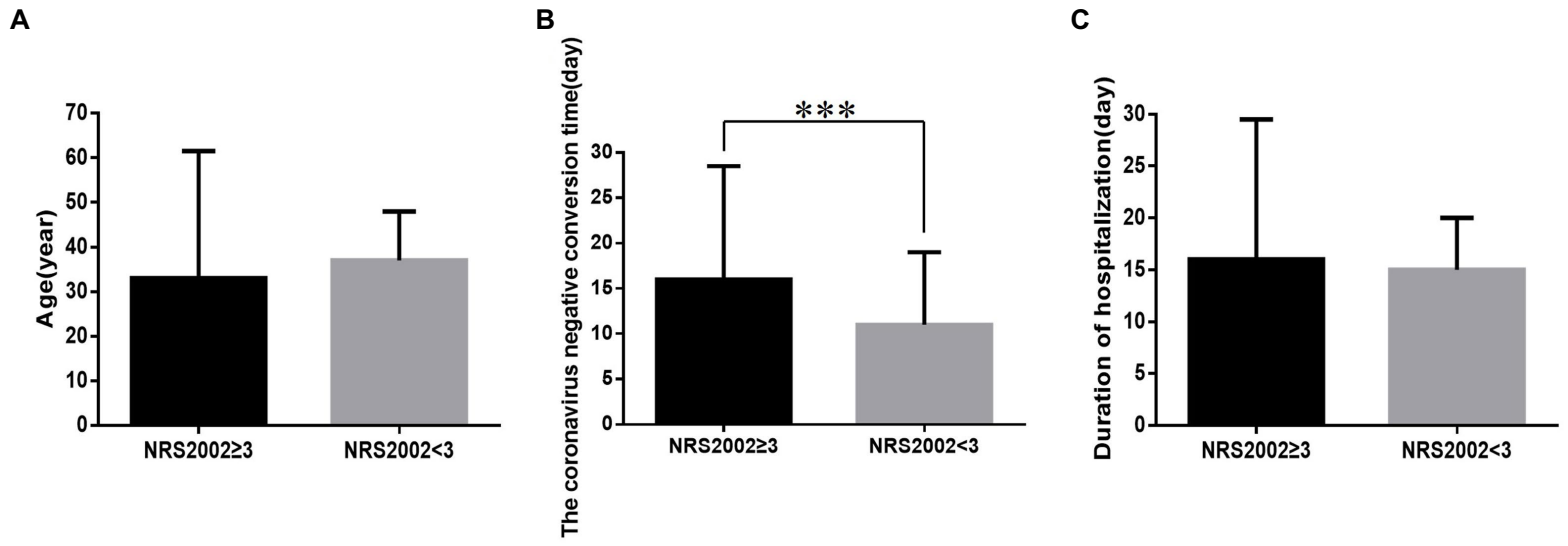
Comparison of age, the coronavirus negative conversion time and duration of hospitalization between NRS2002 ≥ 3 group and NRS2002 < 3 group (n = 1,228; NRS2002 ≥ 3 and NRS2002 < 3 groups, *n* = 97 and 1131, respectively). **(A)** Age. **(B)** The coronavirus negative conversion time. **(C)** Duration of hospitalization. All variables were presented as median (IQR). Wilcoxon rank-sum tests were used for inter group comparison. (**A,C**, *p* all>0.05; **B**, *p* < 0.001) ***p < 0.001.

In addition, in the with-nutritional risk group, the coronavirus negative conversion time (Figure 1B) was significantly higher in the without-nutritional risk group (all *p* < 0.05). However, there were no statistically significant differences in age (Figure 1A) or duration of hospitalization (Figure 1C) between these two groups (all *p* > 0.05).

**Figure 2:**
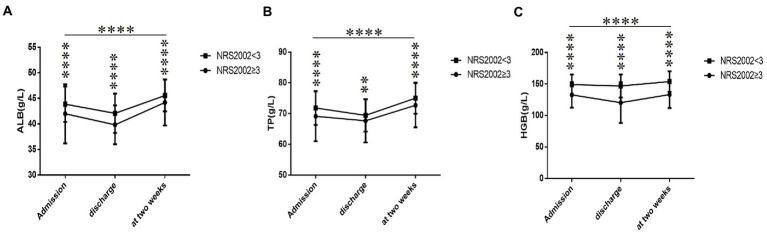
Comparison of ALB, TP and HGB between NRS2002 ≥ 3 group and NRS2002 < 3 group within 2 weeks (*n* = 1187; NRS2002 ≥ 3 and NRS2002 < 3 groups, *n* = 92 and 1095, respectively). ALB, albumin; TP, total protein; HGB, hemoglobin. **(A)** ALB. **(B)** TP. **(C)** HGB. All variables were presented as mean. Unpaired two ANOVA were used for intergroup comparison. **p < 0.01, ****p < 0.0001.

Meanwhile, whether with or without the nutritional risk group, ALB level (Figure 2A), TP level (Figure 2B), and HGB level (Figure 2C) declined rapidly after admission to low values at discharge, then gradually raised to the peak at 2 weeks. Compared with the without-nutritional risk group, whether admission, discharge or 2 weeks, the ALB level (Figure 2A), TP level (Figure 2B), and HGB level (Figure 2C) were significantly lower in the with-nutritional risk group (all *p* < 0.05).

### 3.3. The impact of nutritional risk and NRS2002 score on disease severity and prognosis in patients with COVID-19

Spearman correlation analysis showed that age, sex, clinical type of COVID-19, number of comorbidities, coronavirus negative conversion time, duration of hospitalization, with-nutritional risk and NRS2002 score were positively related to disease severity, while source of cases, ALB level, TP level and HGB level at admission were negatively related to disease severity (Table 3). Factors positively associated with prognosis were age, sex, clinical type of COVID-19, number of comorbidities, with-nutritional risk and NRS2002 score, while source of cases, ALB level, TP level and HGB level at admission were negatively related to prognosis (Table 3).

**Table 3:** Spearman correlation analysis between nutritional parameters, nutritional risk, NRS2002 score and the disease severity and prognosis (*n* = 1,228).

Variables	Disease severity (1 = critical, 0 = non-critical)		Prognosis (1 = cured, 2 = death)	
	*r*	*p*	*r*	*p*
Age (year)	0.207	<0.001	0.108	<0.001
Sex	0.061	0.033	0.061	0.033
Clinical type of COVID-19	0.357	<0.001	0.121	<0.001
Number of comorbidities	0.209	<0.001	0.085	0.003
Source of cases	−0.399	<0.001	−0.167	<0.001
The coronavirus negative conversion time (day)	0.163	<0.001	–	–
Duration of hospitalization (day)	0.068	0.017	–	–
Nutritional risk	0.496	<0.001	0.218	<0.001
NRS2002 score	0.513	<0.001	0.183	<0.001
ALB at admission (g/L)	−0.199	<0.001	−0.107	<0.001
TP at admission (g/L)	−0.181	<0.001	−0.101	<0.001
HGB at admission (g/L)	−0.157	<0.001	−0.100	<0.001

NRS2002, nutritional risk screening 2002; ALB, albumin; TP, total protein; HGB, hemoglobin.

In addition, using Spearman’s partial correlation coefficient analysis, the correlations remained significant after controlling for age and number of comorbidities (Table 4).

**Table 4:** Spearman’s partial correlation analysis between nutritional parameters, nutritional risk, NRS2002 score and the disease severity and prognosis after controlling for age and number of comorbidities (*n* = 1,228).

Control variables	Variables	Disease severity (1 = critical, 0 = non-critical)		Prognosis (1 = cured, 2 = death)	
		*r*	*p*	*r*	*p*
Age	Sex	0.070	0.014	0.058	0.044
Number of comorbidities	Clinical type of COVID-19	0.331	<0.001	0.112	<0.001
	Source of cases	−0.327	<0.001	−0.112	<0.001
	The coronavirus negative conversion time (day)	0.135	<0.001	–	–
	Duration of hospitalization (day)	0.089	0.002	–	–
	Nutritional risk	0.493	<0.001	0.204	<0.001
	NRS2002 score	0.635	<0.001	0.349	<0.001
	ALB at admission (g/L)	−0.187	<0.001	−0.109	<0.001
	TP at admission (g/L)	−0.181	<0.001	−0.110	<0.001
	HGB at admission (g/L)	−0.196	<0.001	−0.171	<0.001

NRS2002, nutritional risk screening 2002; ALB, albumin; TP, total protein; HGB, hemoglobin.

The risk factors for disease severity by multiple linear regression analysis were with-nutritional risk, NRS2002 score, source of cases, clinical type of COVID-19 and ALB at admission level (Table 5). Moreover, the risk factors for prognosis were nutritional risk, NRS2002 score and TP at admission level (Table 5).

**Table 5:** Multiple stepwise regression analysis of influencing factors of the disease severity and the prognosis (*n* = 1,228).

Independent variables		*B*	Std. error	Beta	*t*	*p*
Disease severity	Constant	1.141	0.052	–	2.709	0.007
	Nutritional risk	−0.353	0.034	−0.512	−10.373	<0.001
	NRS2002 score	0.179	0.009	1.042	19.763	<0.001
	Source of cases	−0.042	0.012	−0.076	−3.474	0.001
	Clinical type of COVID-19	0.029	0.004	0.155	7.217	<0.001
	ALB at admission (g/L)	−0.003	0.001	−0.061	−3.026	0.003
Prognosis	Constant	1.038	0.020	–	52.216	<0.001
	Nutritional risk	−0.180	0.014	−0.760	−12.517	<0.001
	NRS2002 score	0.063	0.004	1.064	17.368	<0.001
	TP at admission (g/L)	−0.001	0.000	−0.055	−2.157	0.031

NRS2002, nutritional risk screening 2002; ALB, albumin; TP, total protein.

### 3.4. Role of the NRS2002 score in predicting disease severity and prognosis in patients with COVID-19

**Figure 3:**
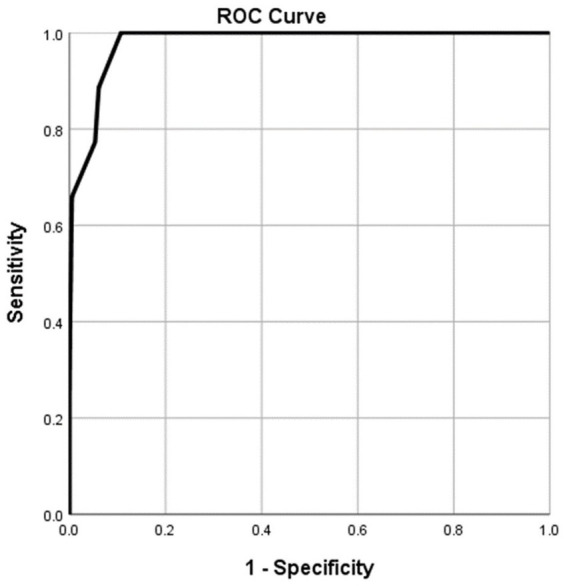
Using characteristics of NRS2002 score for discriminating the critical cases from the non-critical patients (*n* = 1,228; critical and non-critical groups, *n* = 44 and 1,184, respectively). ROC analysis showing the performance of NRS2002 score in distinguishing critical cases from non-critical patients. The parametric estimate of the area under the ROC curve and its 95% confidence interval are 0.980 and 0.970 ~ 0.990, respectively. ROC, receiver operating characteristic curve; AUC, area under the curve.

**Figure 4:**
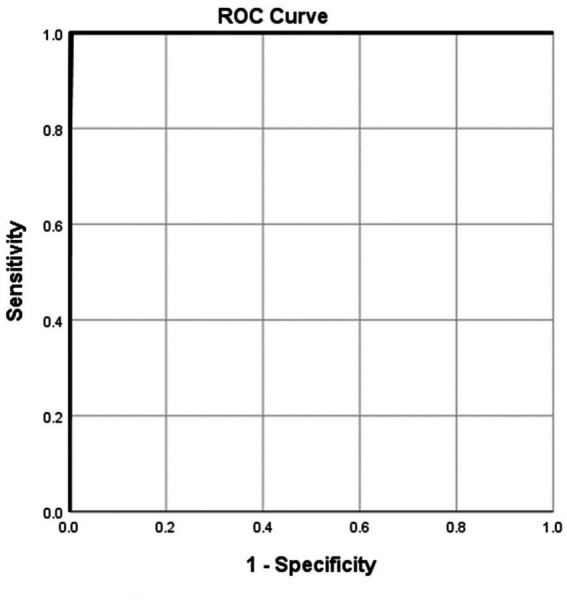
Using characteristics of NRS2002 score on admission for discriminating the surviving cases from the dead patients (*n* = 1,228; surviving and dead groups, *n* = 1223 and 5, respectively). ROC analysis showing the performance of NRS2002 score in distinguishing the dead cases from the surviving patients. The parametric estimate of the area under the ROC curve and its 95% confidence interval are 0.999 and 0.998 ~ 1.000, respectively. ROC, receiver operating characteristic curve; AUC, area under the curve.

According to the ROC analysis, the NRS2002 score showed good utility for predicting critical COVID-19 patients (Table 6) and dead COVID-19 patients (Table 7). The areas under the curve were 0.980 (Table 6; Figure 3) and 0.999 (Table 7; Figure 4), respectively. The thresholds were 0.5 and 5.5, respectively. The sensitivities were all 100.00%. The specificities were 89.30 and 99.60%, respectively (Tables 6, 7).

**Table 6:** The performance of various methods for distinguishing between severe cases and non-severe cases (*n* = 1,228).

Variable	Cutoff point	AUC (95% CI)	Sensitivity	Specificity	False positive	False negative
NRS2002 score	0.5	0.980 (0.970 ~ 0.990)	100.00%	89.30%	0.00%	10.70%

NRS2002, nutritional risk screening 2002; AUC, area under the curve; CI, confidence interval.

**Table 7:** The performance of various methods for distinguishing between cured and death (*n* = 1,228).

Variable	Cutoff point	AUC (95% CI)	Sensitivity	Specificity	False positive	False negative
NRS2002 score	5.5	0.999(0.998 ~ 1.000)	100.00%	99.6%	0.00%	0.4%

NRS2002, nutritional risk screening 2002; AUC, area under the curve; CI, confidence interval.

## 4. Discussion

In this retrospective study, we reported patients at nutritional risk were 7.90% with NRS2002. Nutritional risk, NRS2002 score and ALB at admission were risk factors for disease severity. Moreover, nutritional risk, NRS2002 score and TP at admission were risk factors for prognosis. The NRS2002 score showed the best utility for predicting critical illness and death in COVID-19 patients.

Nutritional risk refers to the risk of adverse clinical outcomes from existing or potential nutritional and metabolic conditions (20). COVID-19 is a highly contagious disease. Severe cases are often combined with other organ dysfunctions and are prone to malnutrition (21). Nutritional status is crucial to the maintenance of the body’s immune function. Malnutrition not only impairs immune defense mechanisms, but may also increase susceptibility to infection (22). In addition, malnutrition can be the result of infection (23). Thus, reasonable nutritional support can timely prevent the increase in the incidence of multiple organ failure, improve the patient’s immune function, shorten the course of disease, and reduce the mortality rate (5). Nutritional risk screening is the first step in nutritional support. Studies have demonstrated that NRS2002 has higher sensitivity than other traditional nutritional screening tools (24). In this study, we found that 7.9% of COVID-19 patients had nutritional risk. Previous studies have shown that 82.6–92% of COVID-19 patients are at nutritional risk (25–27). In the literature, 9.96% of COVID-19 patients at nutritional risk were closest to our findings (28). The inconsistency may be because most of the patients in this study were non-critical, while only 44 were critical, and the median age was only 37 years, with a predominance of young people in the onset population and a larger sample size. Aged 65 years and older have been shown to be an important risk factor for deterioration and death in COVID-19 patients (29, 30). Patients with more severe disease are more likely to have nutritional risk (31).

In previous studies with a median age of 60 years, patients with COVID-19 at nutritional risk were older than those without nutritional risk (25). Elderly patients are more prone to reduce nutrient absorption due to symptoms such as loss of appetite and decreased intestinal function, combined with the combination of COVID-19, inadequate intake due to stress and fear (32), compensatory effects of hypoxia on vital organs, and gastrointestinal reactions such as nausea, vomiting, and anorexia caused by some medications during treatment, making these patients more susceptible to nutritional risk (33). Patients with COVID-19 who are at nutritional risk are more likely to develop complications and have longer hospital stays (34). In contrast, our findings are inconsistent with this finding. COVID-19 patients with nutritional risk were younger and had a longer duration of hospitalization than those without nutritional risk, but the differences were not statistically significant. The reason may be related to the median age of 37 years in our study, which was predominantly young, and the low proportion of patients with nutritional risk, which was only 7.90%.

Meanwhile, we found that COVID-19 patients with nutritional risk had a longer time to be coronavirus negative than those without nutritional risk. Nutrition plays a key role in improving immunity. For viral infectious diseases, nutritional status affects the viral genome mutation from benign or minimally pathogenic viruses to highly pathogenic viruses and their transmission in the host (35). If there is a nutritional risk, it can directly affect the immune defense.

ALB, TP and HGB are commonly used in clinical practice as indicators of malnutrition. Studies have suggested that ALB and HGB have a negative correlation with the NRS2002 score (36). This study showed that whether admitted, discharged or at 2 weeks, ALB level, HGB level, and TP level were significantly lower in patients with nutritional risk than in those without nutritional risk and that ALB at admission level was a significant factor for disease severity. This finding is consistent with literature conclusions (31, 37) that ALB is a reliable indicator of nutritional status and correlates with the prognosis of the severity of COVID-19 (38). Low ALB level indicates nutritional deficiencies or an organism in a state of intense stress (39). We speculate that the lower ALB in COVID-19 patients with nutritional risk may be due to reduced protein synthesis and increased consumption due to poor appetite, stressful conditions, and more comorbidities in patients.

In addition, this study found that in the with-nutritional risk group, the proportion with three or more comorbidities was significantly larger, and the rates of critical illness and mortality were higher than those in the without-nutritional risk group. Similarly, nutritional risk has an important influence on disease progression and prognosis in COVID-19 patients. The more comorbidities the patients have and the more serious their conditions are, the more likely they are to have impaired organ function (40), and coupled with the fact that patients are severely underfed or unable to eat, have disrupted catabolism, or even have viruses directly invading the digestive system to impede nutrient absorption (41), they are more prone to malnutrition and have a greatly increased probability of nutritional risk. This results in further impairment of the patient’s immune function and contributes to the progression from asymptomatic infection, light, and common to severe and critical forms, thus causing a poor prognosis.

Previous studies have shown that the NRS2002 score can be an appropriate and practical predictor of prognosis for COVID-19 patients (26, 42), an independent predictor of the clinical type of COVID-19 patients (28), and indirectly reflecting the severity and prognosis of COVID-19 (43). In this study, the NRS2002 score not only had a significant impact on disease progression and prognosis in COVID-19 patients but also had a good predictive value for both disease severity and prognosis, with cutoff values of 0.5 and 5.5, respectively. The higher the NRS2002 score is, the greater the risk of critical illness and the worse the prognosis of COVID-19 patients. Therefore, early screening for nutritional risk in patients with COVID-19 is crucial. Reasonable nutritional support is extremely important for patients with severe COVID-19. Infections can be better controlled with nutritional support, improving the patient’s prognosis.

However, there are still some limitations of this study. Importantly, it was a single-center, retrospective study, and all the inherent limitations of retrospective studies are unavoidable and do not allow for causal inference. The number of severe cases, especially deaths, was small. Moreover, we did not consider the association of dietary habits, mental condition, and social support with the nutritional status of patients. We did not systematically collect data on patient-specific dietary intake, malnutrition diagnosis and nutritional support. We realized that this information may be useful from a clinical point of view in the management of COVID-19 patients.

## 5. Conclusion

The results of the present study indicated that both the with-nutritional risk and NRS2002 score are important influencing factors of COVID-19 disease severity and prognosis. COVID-19 disease severity and prognosis were positively correlated with nutritional risk and NRS2002 score but negatively correlated with ALB level, TP level, and HGB level at admission. In addition, the NRS2002 score has good predictive value for disease progression and poor prognosis. For patients with NRS2002 score > 0.5, should be closely monitored and appropriate early interventions can be made to reduce the occurrence of critical diseases. For patients with NRS2002 score > 5.5, nutritional support treatment should be actively given to reduce mortality and improve prognosis. Furthermore, our findings further point to the importance of rapid nutritional screening after hospital admission in patients with COVID-19 and suggest that further research in this area to determine early nutritional needs may help improve disease prognosis.

## Data availability statement

The raw data supporting the conclusions of this article will be made available by the authors, without undue reservation.

## Ethics statement

The studies involving human participants were reviewed and approved by the Ethics Committee of the Public and Health Clinic Centre of Chengdu (ethics approval number: PJ-K2020-26-01). The patients/participants provided their written informed consent to participate in this study.

## Author contributions

YuZ, DL, YC, and YaZ: concept and design. YuZ, BZ, and FG: data acquisition. YuZ, DL, and YaZ: data analysis and interpretation. YuZ, ZX, DL, and YaZ: drafting the manuscript. DL, YC, YaZ, BZ, FG, and XY: administrative, technical, or material support. DL and YaZ: study supervision. All authors contributed to the article and approved the submitted version.

## Funding

This research was supported by the Thirteenth Five-Year Project on Tackling Key Problems of National Science and Technology (2017ZX10305501008), the Nonprofit Central Research Institute Fund of the Chinese Academy of Medical Sciences (2020-PT330-005), the Sichuan Science and Technology Program (2020YFS0564), The Chengdu Municipal Science and Technology Bureau Science and Technology Huimin Major Demonstration Project (00092), the Sichuan Province Health Commission (17PJ070), the Chengdu Municipal Health Commission (2019079), and the Chengdu Science and Technology Bureau (2021-YF05-00536-SN).

## Conflict of interest

The authors declare that the research was conducted in the absence of any commercial or financial relationships that could be construed as a potential conflict of interest.

## Publisher’s note

All claims expressed in this article are solely those of the authors and do not necessarily represent those of their affiliated organizations, or those of the publisher, the editors and the reviewers. Any product that may be evaluated in this article, or claim that may be made by its manufacturer, is not guaranteed or endorsed by the publisher.

## Abbreviations

COVID-19: Coronavirus Disease 2019
SARS-CoV-2: Severe Acute Respiratory Syndrome Coronavirus 2
ESPEN: European Society for Clinical Nutrition and Metabolism
NRS2002: Nutrition Risk Screening 2002
RT-PCR: Reverse Transcription-Polymerase Chain Reaction
ICU: Intensive Care Unit
BMI: Body Mass Index
ROC: Receiver Operating Characteristic
ALB: Serum Albumin
TP: Total Serum Protein
HGB: hemoglobin
